# Teaching Student Veterinary Professionals About Pharmacovigilance: A Survey of UK Veterinary Educators

**DOI:** 10.1111/jvp.70054

**Published:** 2026-02-26

**Authors:** Heather Grieve, Martin Hawes, Catherine Hemingway, Elizabeth Wright, David Killick

**Affiliations:** ^1^ Institute of Infection, Veterinary and Ecological Sciences University of Liverpool Liverpool UK; ^2^ Department of Comparative Biomedical Sciences, School of Veterinary Medicine University of Surrey Guildford UK; ^3^ The College of Animal Welfare The College of Animal Welfare Huntingdon UK; ^4^ The Royal (Dick) School of Veterinary Studies University of Edinburgh Edinburgh UK

**Keywords:** adverse drug reaction, adverse event, pharmacology, pharmacovigilance, survey, veterinary education, veterinary medicine, veterinary nursing

## Abstract

Adverse drug event (ADE) reports submitted by veterinary professionals are essential for monitoring the safety and efficacy of marketed veterinary products. Despite this, the majority of veterinary ADEs are not reported. Further education on the topic of pharmacovigilance has been widely explored in human healthcare as a potential facilitator for reporting. To guide design of an enhanced pharmacovigilance curriculum for veterinary professionals, a better understanding of student veterinary professional pharmacovigilance training is required. Therefore, we investigated the current UK pharmacovigilance curriculum through a survey of veterinary educators. In total, there were 17 responses from UK‐based training providers, six relating to undergraduate veterinary surgeon training and 11 relating to training in veterinary nursing. All students received some pharmacovigilance training, which appeared to be largely didactic in nature. A total of 29.4% of respondents indicated that students are provided the opportunity to submit a practice ADE report. Exploration of the knowledge, attitudes, and perceptions of students with regards to this training would be useful in assessing quality and opportunities for enhancement. Development of shared resources which provide students with access to ‘real world’ examples of ADEs would support development of practical skills in identifying, managing, and reporting ADEs.

## 1 Introduction

All veterinary medicinal products (VMPs) have the potential to cause adverse drug events (ADEs). ADEs, often referred to as adverse events or adverse drug reactions depending on context, are defined as ‘any observation in animals, whether or not considered to be product‐related, that is unfavourable and unintended and that occurs after any use of VMP (off‐label and on‐label uses)’ (International Cooperation on Harmonisation of Technical Requirements for Registration of Veterinary Medicinal Products 2007). This also encompasses events related to a suspected lack of expected efficacy according to approved labelling or noxious reactions in humans or the environment. Where there is reasonable probability that an ADE was caused by a VMP, then it may be concluded that the ADE was an adverse drug reaction. ADEs are identified, monitored, and mitigated through the pharmacovigilance process, which helps to ensure that the overall benefit–risk evaluation for a product holding a marketing authorization remains favourable. As an example, the United Kingdom's (UK) regulator for VMPs, the Veterinary Medicines Directorate (VMD), received and assessed 6139 ADE reports in 2020 and updated the product information for 88 VMPs based on information received (Veterinary Medicines Directorate 2022), thus demonstrating the importance of report submission for the ongoing monitoring of the safety and efficacy of VMPs.

Veterinary professionals play a central role in pharmacovigilance through the submission of so‐called ‘spontaneous’ ADE reports arising from observations within their clinical practice. Reports can be submitted to medicines regulators (the ‘national competent authority’ [NCA]) or to the pharmaceutical company responsible for the VMP (the ‘marketing authorisation holder’ [MAH]), and these organisations share submitted reports with each other. Under current UK legislation at the time of writing, MAHs must submit all valid ADEs relating to VMPs marketed in the UK to the VMD, regardless of where the ADE occurred (Veterinary Medicines Directorate 2024). Veterinary professionals in the UK are encouraged to report ADEs by the Royal College of Veterinary Surgeons (RCVS) Code of Professional Conduct (Royal College of Veterinary Surgeons 2024a, 2024b). Moreover, awareness of how to report is listed amongst the Day One Competences for both veterinary surgeons (Royal College of Veterinary Surgeons 2022a) and veterinary nurses (Royal College of Veterinary Surgeons 2022b). Despite this, a growing body of evidence suggests that most ADEs are not reported (Fresnay et al. 2015; Sander et al. 2020; Mount et al. 2021; Wilson et al. 2021; Davies et al. 2024).

The issue of underreporting is not unique to veterinary medicine, with a similar underreporting rate suggested in human healthcare (Hazell and Shakir 2006). Unsurprisingly, there are many examples in the published literature of studies seeking to improve the reporting rate in human healthcare through a variety of interventions such as appointing a person responsible for ADE reporting (Lander et al. 2013; Reumerman et al. 2021b) and electronic reporting tools (Ortega et al. 2008; Barrow et al. 2012). Education is also often posed as a facilitator to ADE reporting. Routledge and Bracchi recently published an overview of the systematic reviews published from 2000 to 2020 which explore ADE reporting interventions (Routledge and Bracchi 2023). They detail that of the 101 studies included in these reviews, 57 include education as the primary intervention, and most studies resulted in an increase in reporting.

Routes to improving the reporting rate of ADEs are poorly explored in veterinary medicine. However, a previous survey of UK veterinary professionals suggested further training is perceived as a facilitator of ADE reporting, especially amongst veterinary nurses (Davies et al. 2022). However, the aforementioned study did not explore what training respondents had received regarding ADE reporting during their undergraduate studies, and as such the potential to improve this training is unknown. Therefore, the aim of this study was to explore the pharmacovigilance training provided to student veterinary surgeons and veterinary nurses via a survey aimed at UK based veterinary educators.

## 2 Methods

A survey was developed and launched using the JISC online survey tool. Questions were developed by two authors with experience in veterinary drug safety (HG & DK) and veterinary education (DK). The survey consisted of a maximum of 17 questions relating to the type of qualification offered by the provider and the pharmacovigilance training delivered (if any) including year of study, details of training format and associated learning outcomes. Participants were also asked if they would be interested in an educational resource to assist with pharmacovigilance training, and suggestions for the type and length of this resource. Question styles included closed questions (multiple choice) and open questions (free text). Open questions were used to allow respondents to clarify (i.e., where they had selected ‘other’), and to collect information regarding learning outcomes. Full details of the survey questions are available in Appendix S1. The survey was approved by the University of Liverpool Veterinary Research Ethics Committee prior to survey launch (VREC1330).

A link to the survey and a brief description of the study was distributed to all veterinary schools (*n* = 10) and veterinary nursing training providers (*n* = 66) in the UK via email. Training providers were identified from the list published by the RCVS which includes details of the accredited courses in the UK (Royal College of Veterinary Surgeons 2024c, 2024d, 2024e). Contact details for pharmacology educators, programme directors or those involved in curriculum planning were identified online. The email encouraged recipients to forward the survey to the most appropriate staff member if the recipient felt they were not able to fully answer questions about pharmacovigilance training. The survey was also posted to the Veterinary Educators in Pharmacology Special Interest Group (VEPSIG) website discussion board. The survey was open from 03‐Jul‐2023 and closed on 25‐Sep‐2023. Due to the less‐than‐ideal number of responses received by veterinary schools, the survey was reopened on 06‐03‐2025 until 03‐04‐2025. Veterinary schools were re‐contacted via email, and the survey was posted to the VEPSIG discussion board.

Survey responses were analysed descriptively. For closed questions, descriptive statistics were produced using Microsoft Excel (2016). Free‐text responses relating to learning objectives were categorised as either practical, theoretical, or drug specific.

## 3 Results

A total of 23 responses were received (17 respondents during the first period that the survey was open, and six during the second). One institution submitted two responses during the first response period, and one institution submitted a response during both the first and second response periods. For these institutions the most complete response was retained. Therefore, the total number of responses available for analysis was 21.

Eleven of the 21 responses were from veterinary nursing training providers teaching the following qualifications: foundation degree in veterinary nursing (*n* = 1), bachelor's degree in veterinary nursing (*n* = 4), Level 3 diploma in veterinary nursing (advanced apprenticeship route) (*n* = 4) and Level 3 diploma in veterinary nursing (non‐apprenticeship route) (*n* = 2). Each of these qualifications leads to the same endpoint (i.e., entry onto the RCVS register of veterinary nurses) therefore responses have been grouped when comparing responses to those received from veterinary medicine training providers. Ten responses were received relating to undergraduate degrees in veterinary medicine, of which four were from outside the UK (two from EU countries and two from non‐EU countries). As pharmacovigilance regulations and professional obligations vary per region, the non‐UK responses are not included in the main portion of the analysis, but they are referred to throughout as an informal comparison.

The response rate, calculated based on the number of institutions contacted for each of the three main groups of award type, was 60.0% (*n* = 6/10) for UK‐based undergraduate veterinary surgeon training providers, 26.3% (*n* = 5/19) for training providers offering degrees in veterinary nursing (foundation and bachelor's) and 12.8% (*n* = 6/47) for training providers offering the Level 3 diploma in veterinary nursing (apprenticeship and non‐apprenticeship pathways).

All respondents indicated that pharmacovigilance training is included in the curriculum. In the UK, training was most frequently provided to student veterinary nurses in the first year or second year of instruction (*n* = 7 and *n* = 5, respectively). For veterinary surgeons, training was most frequently provided in year three (*n* = 4), with no respondents indicating that any pharmacovigilance training is provided in the first year. Overall, 47.1% of respondents (*n* = 8) indicated that pharmacovigilance is covered in more than 1 year of their curriculum. Notably, for the veterinary nursing courses which provide pharmacovigilance training in the first year of training, 42.9% (*n* = 3) provide further training at a later stage in the course. Four (66.7%) of the veterinary surgeon training providers included pharmacovigilance training during more than 1 year of the course.

For almost all programs, training is provided by an internal staff member. Responses from three veterinary surgeon undergraduate courses indicated that an external provider is involved in delivering training (two UK‐based institutions and one outside the UK). The UK‐based institutions indicated that the VMD provides this training. For internal members of staff directly involved in training provision in the UK, their experience of pharmacovigilance was largely garnered from working in clinical practice (100%, *n* = 17), with three individuals also having experience in the pharmaceutical industry, three having experience in regulation, and six having academic and/or research experience.

When asked about the number of sessions relating to pharmacovigilance, for veterinary surgeon training providers one respondent indicated that they did not know the total number of sessions. For the remainder, training was delivered over a single session (*n* = 2), two sessions (*n* = 1) or three sessions (*n* = 2). For veterinary nursing programs, the total number of sessions relating to pharmacovigilance delivered over the whole program was reported as one (*n* = 3), two (*n* = 3), three (*n* = 2) to four or more (*n* = 1). Two training providers did not know the total number of sessions over which pharmacovigilance training was delivered. In terms of total training time, veterinary surgeon training providers indicated that less than 1 h (*n* = 1), 1–2 h (*n* = 2), 3–4 h (*n* = 2) and 5+ h (*n* = 1) were given for this training. The majority of veterinary nursing training providers (54.5%, *n* = 6) indicated that total training time was 1–2 h, whilst 25.7% (*n* = 3) indicated 3–4 h and 9.1% (*n* = 1) provided over 5 h of training. One veterinary nursing training provider indicated that they did not know the total training time.

Almost all UK training providers indicated that training was provided in didactic format, although this was frequently employed in combination with other learning formats. Of the 10 veterinary nurse training providers using didactic learning, 50% (*n* = 5) were also delivering training in small group format, whilst 10% (*n* = 1) were using experiential learning and 10% (*n* = 1) were using a combination of all three. One veterinary nurse training provider reported using small group format only. For veterinary surgeon training providers, 50% (*n* = 3) reported using all three delivery formats, 16.7% (*n* = 1) reported using didactic and small group format, whilst the remainder used didactic training only. Figure 1 depicts the different learning formats reported by training providers to deliver pharmacovigilance training, split by training type (i.e., veterinary surgeon or veterinary nurse).

**FIGURE 1:**
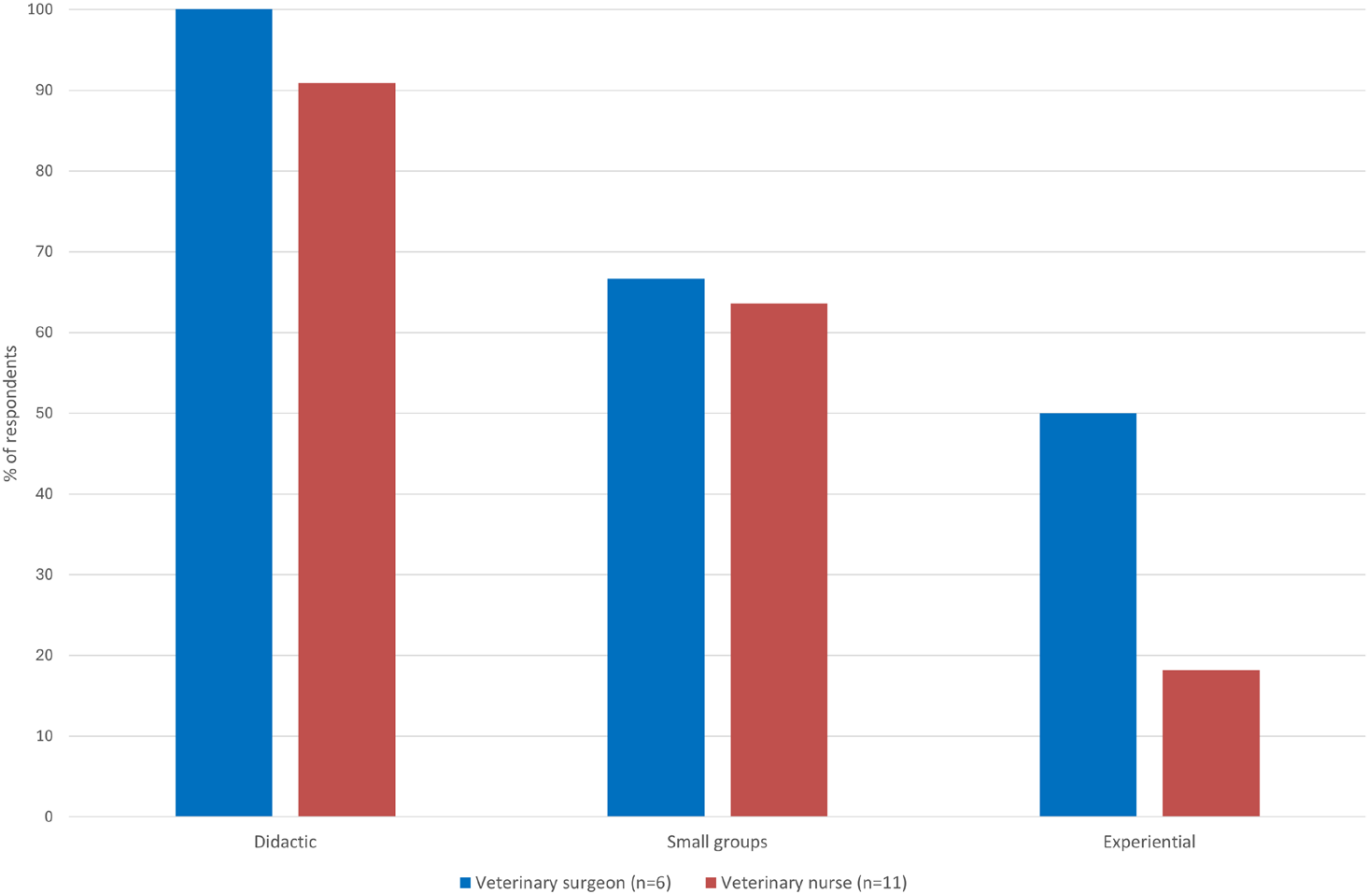
Types of learning used to deliver pharmacovigilance training as indicated by respondents from institutions training undergraduate veterinary surgeons (*n* = 6) and student veterinary nurses (*n* = 11) in the UK. Note that respondents could select multiple learning formats.

A total of 33 learning outcomes or objectives were reported which were grouped into three broad categories; practical understanding (i.e., understanding how to report), theoretical understanding (i.e., understanding why reporting is important) and drug specific (i.e., issues relating to specific drug classes). Example learning objectives and learning outcomes as indicated by respondents are shown in Table 1, separated by student type (i.e., veterinary surgeon or veterinary nurse).

**TABLE 1:** Example learning objectives and outcomes for pharmacovigilance training provided to student veterinary surgeons and veterinary nurses in the UK, grouped into practical understanding, theoretical understanding and drug specific objectives or outcomes.

Outcome/objective category	Example quotation
*Veterinary surgeon*	
Practical understanding (*n* = 5)	‘Describe how to deal with a suspected adverse event and make an official report’ ‘Describe the procedure to report an adverse event’
Theoretical understanding (*n* = 9)	‘Describe the role of the veterinary surgeon in ensuring responsible drug use’ ‘Describe the different types of adverse events’
Drug specific (*n* = 0)	N/A
*Veterinary nurse*	
Practical understanding (*n* = 4)	‘To be aware of possible adverse drug reactions and how to report correctly’ ‘Summarise the Veterinary Medicines Directorate (VMD) procedure for reporting adverse reactions in veterinary medicines’
Theoretical understanding (*n* = 11)	‘Explain the importance of reporting pharmacovigilance issues’ ‘Explain why we should report and discuss error’ ‘Interpret and apply the legislation governing veterinary medicines in the UK’
Drug specific (*n* = 4)	‘List the possible adverse effects of chemotherapy’

All but two (one veterinary medicine and one veterinary nursing training provider) UK training providers stated that they assessed these learning objectives/outcomes through summative assessment. For eight respondents, summative assessment was the only method of assessment used, whereas three veterinary surgeon training providers (50%) and six veterinary nursing training providers (54.5%) stated that they also used formative assessment.

Two respondents did not know if students were given the opportunity to submit a practice report. Otherwise, 11.8% (*n* = 2) indicated students are always given the opportunity to submit a practice report (both were veterinary surgeon training providers), 17.7% (*n* = 3) reported students are given the opportunity dependent on cases seen in clinical training and 58.8% (*n* = 10) are not given the opportunity to submit a practice report.

Overall, almost all (94.1%, *n* = 16) UK‐based respondents indicated that they would be interested in an open access resource to assist with the delivery of pharmacovigilance training, with 1–2 h being the optimal duration (81.3%, *n* = 13/16). All non‐UK based respondents indicated they would be interested in such a resource. For UK based institutions, quizzes were the most popular options for the format of this training material (*n* = 13/16, 81.3%), whilst a pre‐recorded lecture was the least popular option (*n* = 4, 25.0%). Full details of responses split by area of professional training (i.e., veterinary medicine and veterinary nursing) for UK based respondents is shown in Figure 2. For respondents based outside of the UK the preferred formats indicated were practice submissions (*n* = 3), quizzes (*n* = 2), a pre‐recorded lecture (*n* = 2) and training materials provided to the institution for developing their own session (*n* = 1). Ideal training time ranged from less than an hour (*n* = 2) to 3–4 h (*n* = 1), with one respondent indicating 1–2 h would be preferred.

**FIGURE 2:**
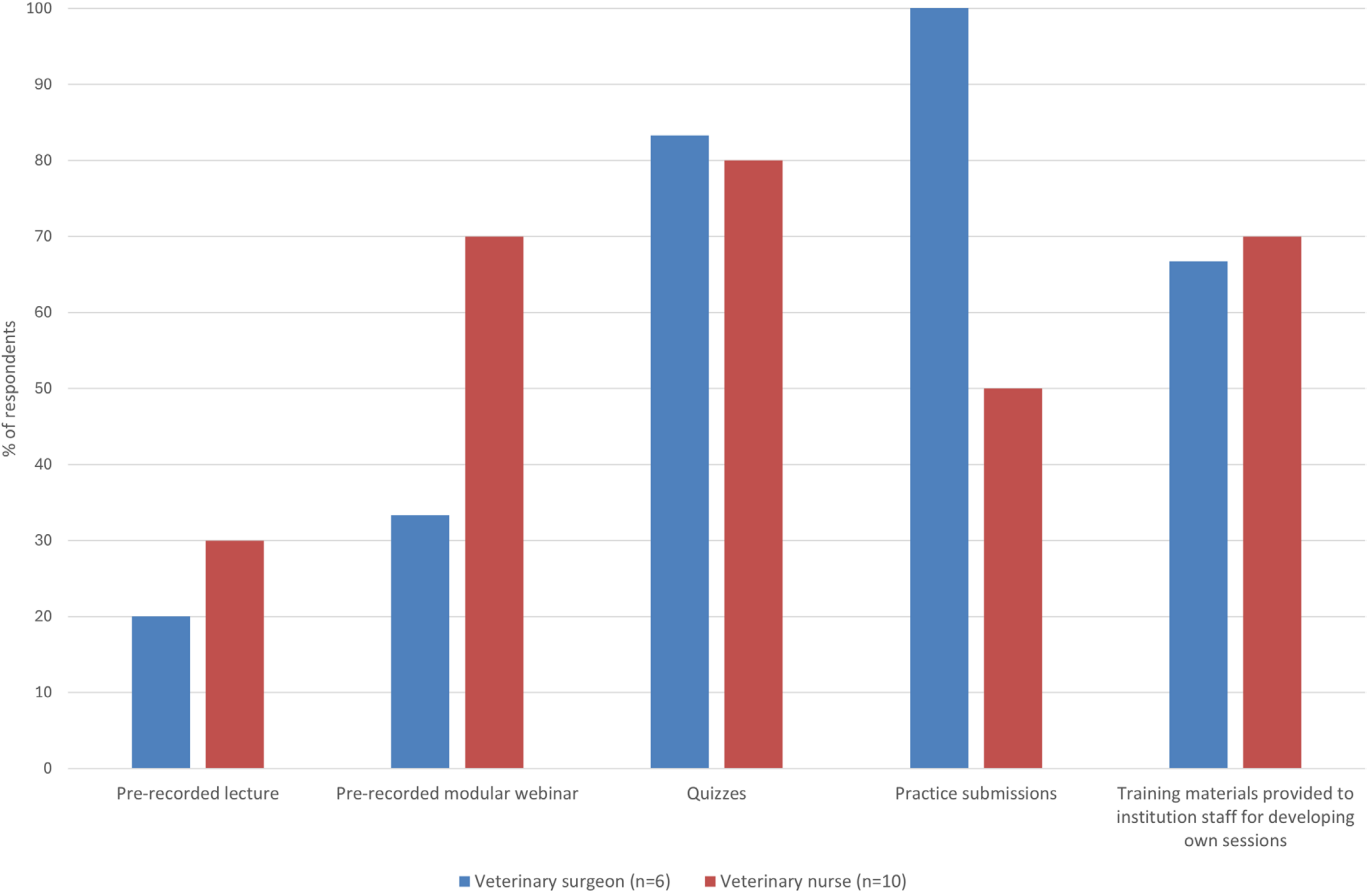
Preferred format for an open access learning resource to assist with delivery of pharmacovigilance training as indicated by respondents from institutions training undergraduate veterinary surgeons (*n* = 6) and student veterinary nurses (*n* = 10) in the UK.

When asked for suggested learning outcomes for this resource, eight respondents provided suggestions. The most common theme was that learning should highlight the importance of pharmacovigilance. Examples included ‘Recognise the importance of pharmacovigilance’, ‘Professional, legal and ethical importance of reporting pharmacovigilance issues’ and ‘Pharmacovigilance and the importance of reporting’. Other suggestions focused on the practical element of reporting including ‘giving students an opportunity to go through the journey of the reporting process’ and ‘Something interactive with a short amount of pre‐recorded content delivery as an intro[duction]’.

When asked for any further comments on pharmacovigilance training for student veterinary professions, eight respondents left comments (seven UK, and one outside the UK). The one non‐UK based respondent highlighted the need for any developed training materials to be adaptable, as legislation varies by region. Of the UK based respondents, two provided suggestions for exploring training of other veterinary staff, specifically SQPs (*n* = 1) and practice managers (*n* = 1). One respondent suggested that case study scenarios would be useful, whilst two comments reflected on either their own institution's pharmacovigilance curriculum or professional training more broadly; ‘Although specifically taught in the sessions identified, it is embedded in other clinical modules’ and ‘I personally [sic] think that it is not covered in enough detail within veterinary curricula’. A further respondent suggested that more emphasis should be given to reporting minor ADEs, as in the respondent's experience ADEs are only reported when all other causes are excluded. Lastly, one respondent suggested that pharmacovigilance training should be mandatory continuing professional development (CPD).

## 4 Discussion

Enhanced training is commonly investigated as a potential intervention to increase the reporting rate of ADEs in human healthcare (Routledge and Bracchi 2023). In a previous survey of veterinary professionals, further education was highlighted as a potential facilitator to reporting (Davies et al. 2022). Little is known about the pharmacovigilance training provided to veterinary surgeons and veterinary nurses during their education. Here we investigated the extent to which pharmacovigilance features on the curriculum at UK‐based veterinary medicine and veterinary nursing courses in order to inform development of an enhanced pharmacovigilance training program.

Our results suggest that all student veterinary professionals currently receive pharmacovigilance training during their studies, but for most this is limited to 3–4 h or less over the course of their whole program. This appears to be broadly in line with their counterparts in human healthcare, where it has been reported that globally, medical students receive a median of 4 h, pharmacy students receive 5.5 h, and allied health professionals 3 h of pharmacovigilance training per course (Hartman et al. 2017). It should be noted that these estimates include broader aspects such as clinical trials, quality issues and medication errors. It is unsurprising that the time dedicated to pharmacovigilance training is somewhat limited given the breadth of material that is required to be covered during veterinary training. Indeed, lack of time in the academic schedule has been cited as a barrier for inclusion of other topics in the veterinary curriculum (e.g., interprofessional education; Estrada et al. 2022). There is perhaps an argument that total time spent on training is not as important as the timing of the training itself. Here, we found that training was often focused in the early to mid‐stages of training, although interpretation is somewhat difficult as length of training varies both per profession (i.e., veterinary surgeon or veterinary nurse) and per training provider. It could be argued that the later stages of training, which are often clinically focused, provide an opportunity for emphasise ADE reporting as a professional activity during placements or rotations.

The RCVS Day One Competences for veterinary surgeons states that veterinary professionals should be able to ‘report suspect adverse reactions effectively’ (Royal College of Veterinary Surgeons 2024b). Similarly, the Day One Competences for veterinary nurses states that registered veterinary nurses should ‘be aware of possible side effects and adverse reactions to medications and alert the veterinary surgeon’ (Royal College of Veterinary Surgeons 2022a). The guidance for this competence suggests that newly qualified veterinary nurses should be aware of the VMD procedures for reporting. Conversely, the language contained within the RCVS Code of Professional Conduct is the same for both veterinary surgeons and nurses and states that if professionals become aware of an ADE following use of a VMP they should record what happened and make a report to either the MAH or the VMD (Royal College of Veterinary Surgeons 2024a, 2024b). Despite the professional obligations placed on veterinary professionals with respect to ADE reporting, we found that most respondents indicated that students are not given the opportunity to complete a mock (practice) ADE report during their training. Whilst almost all respondents indicated that they provide didactic instruction, very few reported providing students with experiential learning opportunities. This is also reflected in the learning objectives and outcomes provided by respondents, where there was a greater number of learning outcomes outlining theoretical understanding compared with those that were more practical in nature. Although for student veterinary surgeons learning outcomes were more evenly distributed between practical and theoretical understanding. Exploring whether newly qualified veterinary surgeons and veterinary nurses feel that they have the skills and confidence to report ADEs would be helpful in determining whether current training is sufficient in developing this competency. Understanding student perception on their preparedness to report ADEs is of particular importance given that some studies have reported that medical and pharmacy students do not feel sufficiently prepared (Reumerman et al. 2018).

All students will undergo some form of work‐based clinical training, for example through rotations and placements. This provides an opportunity to implement an experiential learning model for developing competence in reporting ADEs, helping to underpin didactic teaching. In human healthcare, it has been shown that utilizing medical students as ‘junior adverse drug event managers’ assists with the reporting of ADEs whilst providing students with additional learning opportunities in pharmacology (Reumerman et al. 2021a). In an analysis of the reports submitted by junior adverse drug event managers, it was found that they identified ADEs not previously recognised by other healthcare professionals (Reumerman et al. 2021b). Overall, the program was found to significantly increase the number of reports submitted and, importantly, reports submitted by students were found to be well‐documented. Similarly, a further study utilised pharmacy students to report ADEs at a hospital in Canada (Wentzell et al. 2017). These students completed Health Canada's online pharmacovigilance training modules and used this material to deliver a presentation to qualified pharmacists at their hospital outlining the importance of ADE reporting. Qualified pharmacists were encouraged to contact a designated student reporter with ADE reports, and the student reporter then submitted these reports to the national competent authority. An increase in reporting was demonstrated after implementation of student reporters, although it was not found to be statistically significant. However, in a post‐intervention survey pharmacists at the hospital indicated that this program reduced the perceived barriers to ADE reporting and the pharmacy students involved indicated that the program provided them with insights into the ADE reporting process (Wentzell et al. 2017).

In the past, the VMD has offered to provide pharmacovigilance training at veterinary schools, and here two veterinary schools indicated VMD delivers part of their pharmacovigilance curriculum. However, the total number of schools who take up this offer is unknown. Understanding the motivation for not engaging with training offered by the VMD would be useful, especially since the VMD uses the submitted ADE reports in regulatory decision making and is therefore best placed to educate students on how reports contribute to the overall pharmacovigilance efforts and best practice in report completion. Almost all respondents indicated that they would be interested in utilizing an open access resource to assist with pharmacovigilance training. Whilst it would not be feasible for the VMD to provide training for every UK‐based veterinary surgeon and veterinary nursing training provider, a training resource developed with and approved by the VMD may be useful to meet this need. In recent months, the VMD has developed a modular pharmacovigilance e‐learning program and whilst primarily aimed at national competent authorities in other countries, some introductory material may be suitable for providing to institutions to allow them to develop their own training materials, which was indicated as one of the preferred formats for an open access resource.

Elsewhere, there are several examples of suggested curricula for teaching pharmacovigilance to human healthcare professional trainees, for example the British Pharmacological Society's Medical Curriculum (Ross and Maxwell 2012), which may be useful when designing a similar curriculum for veterinary students. Probably the most developed example was produced by the WHO Collaborating Centre for Pharmacovigilance Education. Here, a stakeholder group developed pharmacovigilance competencies for healthcare students, the so‐called ‘WHO Pharmacovigilance Core Curriculum’ (van Eekeren et al. 2018). The five key competencies identified were: (i) understanding the importance of pharmacovigilance, (ii) preventing ADRs, (iii) recognising ADRs, (iv) managing ADRs and (v) reporting ADRs. Notably, the group highlighted that time and resources can be limited and so the curriculum was designed to be embedded into existing courses. This is perhaps already reflected in some responses to the learning outcomes question of our survey, whereby identifying and managing ADEs are discussed in the context of specific drugs and integrated across the curriculum. It is likely that adverse effects of specific drug classes are taught in several modules at various times over the entire training program, therefore providing repeated opportunities to remind students of how to report an ADE. Adapting the WHO Pharmacovigilance Core Curriculum for the veterinary context may provide educators with a basis for developing enhanced training within their own institutions, in fact many respondents here indicated that they would like to receive training materials for developing their own sessions.

Practice submissions also proved to be a popular format for open access learning materials, with a further respondent indicating in free‐text that case study scenarios would be useful. The creation of case study banks consisting of ‘real world’ ADEs would allow for active learning activities such as completing practice ADE reports or small group discussions about management and client communication. These activities could also be used to assess students to highlight areas where further learning would be beneficial. To the best of our knowledge, no such resource currently exists and would require significant effort for an individual to create. However, crowdsourcing has been successfully used in human pharmacovigilance to share pharmacovigilance education resources (Bate et al. 2017) and could be of potential utility here in creating an open access resource where veterinary pharmacology educators and/or the VMD can submit case studies for use by others.

Beyond traditional approaches, one study utilised cinemeducation to teach students about pharmacovigilance, using clips from the film ‘150 milligrams’ which depicts the true story of the discovery of the cardiotoxicity associated with benfluorex (Cambra‐Badii et al. 2020). This activity resulted in an improvement in understanding about adverse drug reactions and pharmacovigilance processes, measured using a pre and postintervention multiple choice assessment. Students also reported high satisfaction with this activity, further highlighting the effectiveness of real‐world examples when teaching students about pharmacovigilance.

An emerging area of interest in pharmacovigilance is the use of electronic health records (EHRs) for identifying ADEs. In previous work, we have shown that veterinary ADEs can be identified in the free‐text clinical narratives recorded by veterinary professionals during consultations (Davies et al. 2024, 2025; Petrou et al. 2025). As there are both technical and regulatory issues to work through before the use of EHRs can be fully adopted into regulatory pharmacovigilance, their use should be seen as complimentary to, rather than a replacement for, spontaneous reporting at this time. Consequently, introducing students to emerging work in this area may affirm the importance of other professional skills such as good record keeping practices and stimulate interest in clinical informatics, but there is a careful balance needed, as premature lauding of the use of EHRs could discourage spontaneous reporting before informatics techniques are suitably developed to fill the gap.

An obvious limitation of our work is the limited number of responses received. It is therefore possible that the institutions who did not respond provide more training than what is described here. However, it could also be argued that those more interested in pharmacovigilance (and hence providing more in‐depth pharmacovigilance training) may be more likely to respond to this survey, especially since the survey was also advertised via VEPSIG, an international forum for veterinary educators in pharmacology. Further, it is possible that the survey did not reach the individual best placed to provide information about pharmacovigilance teaching, or that there is not a singular individual best placed to provide this information as teaching is spread across the curriculum. The later point is especially true given the integrated nature of veterinary teaching. Some training providers outside of the UK responded to the survey after it was advertised via VEPSIG and we have referred to these responses throughout, though, it is difficult to make direct comparisons due to differences in pharmacovigilance legislation and professional expectations between countries. However, knowing how to report adverse events including therapeutic failure to appropriate agencies is recognised as a Day One competence for veterinary graduates (Werners and Fajt 2021) and has been mapped to the Competency Based Veterinary Education framework (Hawes et al. 2024). Expanding the survey results presented here to capture further data about the pharmacovigilance training provided to student veterinary professionals outside of the UK would provide a more robust comparison. Finally, this project focussed specifically on student veterinary surgeons and veterinary nurses and therefore did not collect information about pharmacovigilance training delivered during training for other professionals legally allowed to prescribe and supply veterinary medicines (i.e., pharmacists and suitably qualified persons [SQPs], or CPD training provided to qualified veterinary professionals). Further research is required to understand the pharmacovigilance training provided to this group of professionals.

## 5 Conclusion & Future Work

Student veterinary professionals in the UK are provided with theoretical pharmacovigilance training but this is often not supported by opportunities to develop practical skills in submitting ADE reports. Most training providers would be interested in an open access resource to support them in delivering pharmacovigilance training. Future work will include surveying student veterinary surgeons and veterinary nurses to capture their knowledge, attitudes and perceptions relating to pharmacovigilance training and their confidence in reporting ADEs in practice. Taken together with the current survey, these results will be used to inform development of open access learning materials in conjunction with key stakeholders such as the VMD, MAHs and educational institutions.

## Author Contributions

H.G. and D.K. designed the study and obtained funding. H.G. analysed the results. H.G. wrote the original draft of the manuscript which was reviewed and edited by M.H., C.H., E.W. and D.K.

## Funding

This research was funded by Research England's Public Policy Quality‐Related Fund allocated to the University of Liverpool.

## Ethics Statement

The authors have nothing to report. The survey was approved by the University of Liverpool Veterinary Research Ethics Committee (VREC1330).

## Conflicts of Interest

The authors declare no conflicts of interest.

## Supporting information


**Appendix S1:** jvp70054‐sup‐0001‐AppendixS1.pdf.

## Data Availability

The data that support the findings of this study are available from the corresponding author upon reasonable request.
