# Supplementary material for: Teaching Student Veterinary Professionals About Pharmacovigilance: A Survey of UK Veterinary Educators
Source: J Vet Pharmacol Ther. 2026 Feb 26;49(6):572–9. doi: 10.1111/jvp.70054 (PMC13745291; doi:10.1111/jvp.70054)
Supplement: Supplementary file 1 [file JVP-49-572-s001.pdf]

Veterinary education for pharmacovigilance/adverse drug reactions

What is the name of your institution? ***Note that this information will only be used to link responses given by students in a separate questionnaire to the curriculum information provided in this questionnaire and to identify duplicate responses.***

Veterinary education for pharmacovigilance/adverse drug reactions

Which qualification does your institution offer? ***If your institution offers more than one of the below qualifications, please indicate the qualification for which you are the programme director and complete the survey based upon this qualification.***

- Undergraduate degree in veterinary medicine/veterinary science
- Foundation degree in veterinary nursing
- A bachelors degree in veterinary nursing (e.g. BSc (Hons))
- Level 3 Diploma in veterinary nursing (advanced apprenticeship)
- Level 3 Diploma in veterinary nursing (non-apprenticeship)

Veterinary education for pharmacovigilance/adverse drug reactions

Does the curriculum for this qualification include pharmacovigilance/adverse event reporting training?

- Yes
- No
- Don't know

Veterinary education for pharmacovigilance/adverse drug reactions

In what year of the programme is this training completed? ***Please indicate all that apply***

- ☐ 1
- ☐ 2
- ☐ 3
- ☐ 4
- ☐ 5
- ☐ 6
- ☐ Don't know

Veterinary education for pharmacovigilance/adverse drug reactions

Who provides this training? ***Please indicate all that apply***

- ☐ Internal staff member
- ☐ External provider
- ☐ Don't know

The person leading this training has experience of pharmacovigilance/adverse event reporting from working in: Please indicate all that apply

- ☐ Clinical practice
- ☐ Regulation
- ☐ Pharma/industry
- ☐ Academia e.g research
- ☐ No previous experience
- ☐ Other

If you selected Other, please specify:

If you selected External provider, please specify:

Veterinary education for pharmacovigilance/adverse drug reactions

Across the entire programme, how many sessions are delivered on pharmacovigilance/adverse event reporting?

- ○ Single session
- ○ 2
- ○ 3
- ○ 4 or more
- ○ Don't know

Veterinary education for pharmacovigilance/adverse drug reactions

What is the total duration of these sessions?

- Less than an hour
- 1-2 hours
- 3-4 hours
- 5+ hours
- Don't know

Veterinary education for pharmacovigilance/adverse drug reactions

What is the nature of these sessions? Please indicate all that apply.

- ☐ Didactic
- ☐ Small groups
- ☐ Experiential
- ☐ All of the above
- ☐ Don't know
- ☐ Other

If you selected Other, please specify:

Veterinary education for pharmacovigilance/adverse drug reactions

What are the learning objectives/outcomes for these sessions?

Veterinary education for pharmacovigilance/adverse drug reactions

Are the learning objectives/outcomes from these sessions assessed? Please indicate all that apply.

- ☐ Yes, formatively
- ☐ Yes, summatively
- ☐ No
- ☐ Don't know

Veterinary education for pharmacovigilance/adverse drug reactions

Do students have the opportunity to submit a practice adverse event report?

- Yes, in all cases
- Yes, depending upon cases seen in clinical training
- No
- Don't know
- Other

If you selected Other, please specify:

Veterinary education for pharmacovigilance/adverse drug reactions

If pharmacovigilance/adverse event reporting is not included in the curriculum, please indicate reasons for this? ***Please indicate all that apply***

- ☐ Limited timetable space
- ☐ Low priority topic
- ☐ Not considered pertinent to this group of trainee professionals
- ☐ Expect this to be covered elsewhere
- ☐ Not previously considered
- ☐ Other

If you selected Other, please specify:

Veterinary education for pharmacovigilance/adverse drug reactions

Would your institution be interested in an open access resource to assist with the delivery of pharmacovigilance/adverse event reporting training?

- Yes
- No

Veterinary education for pharmacovigilance/adverse drug reactions

What would be the optimal duration for such a resource?

- Less than an hour
- 1-2 hours
- 3-4 hours
- 5+ hours

Veterinary education for pharmacovigilance/adverse drug reactions

What would be the ideal delivery method for such a resource? Please indicate all that apply.

- ☐ Pre-recorded lecture
- ☐ Pre-recorded modular webinars
- ☐ Quizzes
- ☐ Practice submissions
- ☐ Training materials provided to institution staff for developing own sessions
- ☐ Other

If you selected Other, please specify:

Veterinary education for pharmacovigilance/adverse drug reactions

Do you have any suggestions for learning objectives/outcomes for such a resource?

Veterinary education for pharmacovigilance/adverse drug reactions

Do you have any other comments about pharmacovigilance/adverse event reporting for trainee veterinary professionals?

Veterinary education for pharmacovigilance/adverse drug reactions

Thank you for participating, without your input this research would not be possible.
